# Analysis of the Research Hotspot of Drug Treatment of Tuberculosis: A Bibliometric Based on the Top 50 Cited Literatures

**DOI:** 10.1155/2022/9542756

**Published:** 2022-01-12

**Authors:** Ying Xiong, Jingwen Wei, Yujia Cai, Yang Zhang, Li Feng, Yonggang Zhang

**Affiliations:** ^1^Department of Periodical Press and National Clinical Research Center for Geriatrics, West China Hospital, Sichuan University, Chengdu, China; ^2^Chinese Evidence-Based Medicine Center, West China Hospital, Sichuan University, Chengdu, China; ^3^West China School of Medicine, Sichuan University, Chengdu, China; ^4^Regenerative Medicine Research Center, West China Hospital, Sichuan University, Chengdu, China

## Abstract

**Objective:**

The objective of the current study was to analyze the research hotspot of drug treatment for tuberculosis via top literatures.

**Materials and Methods:**

A retrospective analysis was performed on June 7th, 2021. Literatures were searched on the Web of Science Core Collection to identify the top 50 cited literatures related to drug treatment of tuberculosis. The characteristics of the literatures were identified. The outcomes included authorship, journal, study type, year of publication, and institution. Cooccurrence network analysis and visualization were conducted using the VOS viewer (Version 1.6.16; Leiden University, Leiden, The Netherlands).

**Results:**

The top 50 cited literatures were cited 308 to 2689 times and were published between 1982 and 2014. The most studied drugs were the first-line drugs such as isoniazid and rifampicin (*n* = 22), and drug-resistant tuberculosis was most frequently reported (*n* = 16). They were published in 18 journals, and the *New England Journal of Medicine* published the most literatures (*n* = 18), followed by the *American Journal of Respiratory and Critical Care Medicine* (*n* = 7), and the *Lancet* (*n* = 6). The authors were from 13 countries, and the authors from the USA published most of the literatures (*n* = 30), while authors from other countries published less than five literatures. The CDC in the USA (*n* = 4), the World Health Organization (WHO) (*n* = 3), and the American Philosophical Society (*n* = 3) were the leading institutions, and only two authors published at least two top-cited literatures as first authors.

**Conclusions:**

This study provides insights into the development and most important literatures on drug therapy for tuberculosis and evidence for future research on tuberculosis treatment.

## 1. Introduction

Tuberculosis is the disease with the largest number of deaths caused by a single pathogen in the world. Based on the World Health Organization (WHO) global tuberculosis report, 10 million people were infected with tuberculosis and 1.5 million died in 2018 [1]. Tuberculosis is still the infectious disease that kills most people worldwide, resulting in a large disease burden [1–3]. Tuberculosis is caused by *Mycobacterium tuberculosis*, and it spreads through the air. It destroys the lungs and other systems and organs in the human body, and it forms tubercles, infiltration, caseation, or cavities, resulting in long-term low fever, expectoration, hemoptysis, and even death [4, 5].

Tuberculosis is preventable and treatable, and its main treatment is drug therapy (chemotherapy). There are over ten kinds of antituberculosis drugs in the world. The modern tuberculosis control strategy (directly observed treatment short-course, DOTS) based on short-term chemotherapy was proposed by the WHO. This strategy recommends 4-6 standard antibiotics for 6-8 months of treatment for active and drug-sensitive tuberculosis, and this is still an important way to treat and control tuberculosis [6] until now. The common first-line oral antituberculosis drugs include isoniazid, rifampin, pyrazinamide, and ethambutol [7]. For multidrug-resistant tuberculosis with reduced sensitivity to first-line antituberculosis, second-line drugs (such as p-aminosalicylic acid, ethylisonicotinic acid, cycloserine, and tertizide), injectable antituberculosis drugs (such as streptomycin, kanamycin, amikacin, and capreomycin), and quinolones (such as levofloxacin, moxifloxacin, and gatifloxacin) can be used. For the treatment of extensively drug-resistant tuberculosis, new antituberculosis drugs can also be used, such as the new mechanism and new target antituberculosis drugs that are represented by betaquiline and delamanine, which were recently introduced onto the market [8–12].

Bibliometrics is a cross-science that uses mathematical and statistical methods to quantitatively analyze all knowledge carriers [13]. It has been widely used for quantitative research assessment exercises of academic output [14, 15]. Citation analysis is the main bibliometric method [16], and the number of citations reflects the impact of an article in the scientific community to a certain extent. Highly cited literatures are considered to be the basis of research fields [17, 18]. Therefore, the analysis of highly cited articles can provide information on the scientific progress and research trends within a specific discipline [19]. Currently, many literatures related to citation analysis have been published in areas such as diabetes [20], surgery [21], anesthesia [22], rehabilitation [23], and vaccines [24], and there are some highly cited tuberculosis-related literatures [25, 26]; however, no study on tuberculosis chemotherapy has been published and the research hotspot is still unclear. Thus, we performed the current study to analyze the research hotspot of drug treatment of tuberculosis by analyzing the top literatures.

## 2. Materials and Methods

We performed a study to analyze the published literature on drug treatment of tuberculosis. This study did not involve human patients, and therefore, it did not require institutional review board approval.

### 2.1. Search Strategy

We performed a search for literatures on drug treatment of tuberculosis in the Web of Science Core Collection on June 7th, 2021. The search terms were “TS= (TB OR tuberculosis OR tuberculoses OR Kochs) AND TS= (chemotherapy OR treatment OR therapy OR effective OR effectiveness OR efficacy OR safety OR safe)”. The search results were sorted by citation, and articles that had more citations were ranked higher.

### 2.2. Article Selection

Two authors independently screened the abstracts and full texts to identify the top 50 cited literatures on drug treatment of tuberculosis. Only articles that focused on the subject of drug treatment of tuberculosis were included. A drug treatment of tuberculosis article was defined as any study that focused on drug treatment of tuberculosis. Articles about *in vitro* research, animal experiments, or drug mechanism research were excluded. In addition, literatures that only mentioned drug treatment of tuberculosis but not its main research purpose were also excluded. Disagreements were resolved by discussion.

### 2.3. Data Extraction

Two authors independently extracted data from the top 50 cited literatures. The data that were extracted included the title, abstract, source journal, publication time, article types, number of authors, name of the first author and corresponding author, author affiliation, country, and journal impact factor.

### 2.4. Data Analysis

After summarizing the relevant content, as mentioned above, all of the data were analyzed by using SPSS software [9]. The data were analyzed using the VOS viewer software to show the journals, countries, institutions, authors, and keywords in the research field of tuberculosis drug therapy.

## 3. Results

The top 50 cited literatures are presented in Table 1. The top 50 cited literatures were identified based on their citations. Overall, there were 26,499 citations, with a range of 308 to 2689 citations, while the average was 530 citations and the median was 432 citations. The most frequently mentioned drugs were the first-line drugs (*n* = 22), which were represented by isoniazid and rifampicin, followed by new drugs (*n* = 16). There were 16 literatures related to drug-resistant tuberculosis, nine literatures related to HIV coinfection, seven literatures related to drug therapeutic schedules, six literatures related to adverse drug reactions (mainly about hepatotoxicity), and two literatures related to supportive drugs.

**Figure 1:**
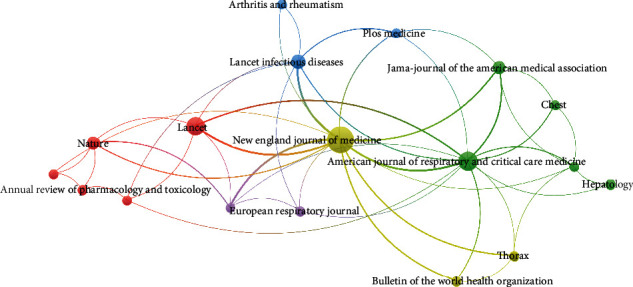
Collaborative network of journals that published the top 50 cited literatures.

These literatures were published in 18 journals (Table 2). Most were published in the *New England Journal of Medicine* (*n* = 18), followed by the *American Journal of Respiratory and Critical Care Medicine* (*n* = 7), *Lancet* (*n* = 6), *Lancet Infectious Diseases* (*n* = 3), *Nature* (*n* = 2), and *JAMA* (*n* = 2). Only one article was published in each of the other 12 journals. The journals' impact factors ranged from 2.268 to 74.699.  Figure 1 shows the collaborative networks of journals that published the top 50 cited literatures. The size of each circle was determined by citations. Additionally, the line in the visualization indicates the relatedness of the journals in terms of cocitation links.

All literatures were published over 33 years, from 1982 to 2014 (Table 3). The highest number of literatures was published in 2003 (*n* = 4), and the maximum contribution of publications was made within a 5-year period from 2010 to 2014 (*n* = 16).

**Figure 2:**
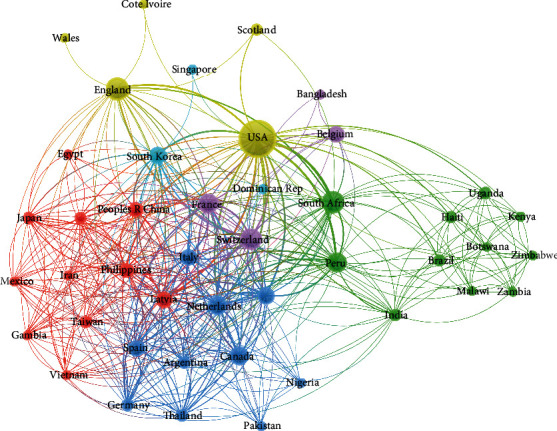
Collaborative network of countries of the top 50 cited literatures.

All literatures were from 13 countries (Table 4). The number of literatures (based on correspondence) from the United States (*n* = 30) accounts for 60% of the total. We obtained the same results by analyzing the source countries of all authors (Table 5) The number of literatures by authors from other countries, including Switzerland, England, Belgium, Canada, South Africa, Spain, China, Denmark, France, the Netherlands, Scotland, and Cote d'Ivoire, was less than five. The difference is that more corresponding authors are from Belgium and more participating authors are from South Africa. From VOS viewer analysis in Figure 2, each node represented a country and lines between the nodes indicated the strength of the relation between countries. The lines' strength of the USA showed a strong connection with others and a great impact on other countries' research.

**Figure 3:**
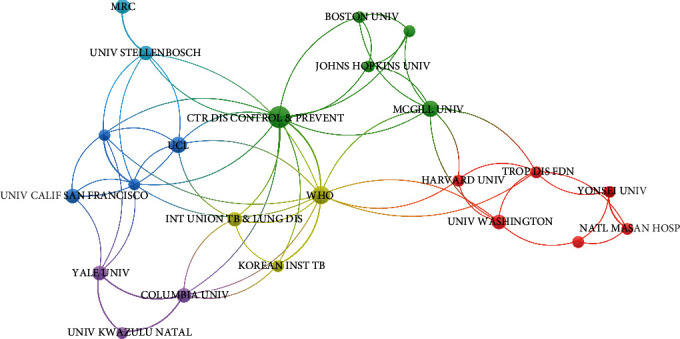
Collaborative network of institutions that published at least two of the 50 top-cited literatures.

Among the 50 literatures, all the authors belong to 195 institutions in total. Five institutions (based on the corresponding author) contributed more than two literatures (Table 6). The five institutions were from three countries, including the USA (*n* = 3), Switzerland (*n* = 1), and South Africa (*n* = 1). The top three contributors were CDC in the USA (*n* = 4), the WHO (*n* = 3), and the American Philosophical Society (*n* = 3). As can be seen from Table 7 and Figure 3, a total of 23 institutions met the threshold of a minimum of 2 publications of the 50 top-cited literatures. It includes 12 American institutions, 3 Korean institutions, 2 South African institutions, 2 British institutions, and Switzerland, Canada, and the Philippines each have one institution. In Figure 3, each node represents an institution; the links represent the association between institutions; and the color and distance between items represent the similarity between institutions. As shown in the network, the Centers for Disease Control and Prevention and WHO linked the most, which meant they had the strongest collaborations with other institutions. Comparing the institutions of the corresponding authors who published more articles with the institutions of all authors, it can be seen that most are Centers for Disease Control and Prevention and the World Health Organization. In addition to the top two institutions, there are some differences between the following institutions, and the participating authors are mostly from different universities.

Among the 50 literatures, there were only two first authors who contributed more than two literatures as the first author. They were Diacon, Andreas H from the University of Stellenbosch in South Africa and Karim, Salim S. Abdool from the University of KwaZulu-Natal in South Africa. No corresponding author published more than one study (Table 8).  Table 9 shows all the authors involved in two or more studies. Among them, Andres, Koen., Iseman, MD., Lounis, and Nacer participated in three studies. These authors are mainly from South Africa (*n* = 9), the United States (*n* = 6), Belgium (*n* = 4), Switzerland, Australia, South Korea, and the United Kingdom (*n* = 1). KwaZulu Natal Union (*n* = 4), Janssen (Pharmaceutical Companies of Johnson & Johnson, *n* = 4), and the Centre for the AIDS Program of Research in South Africa (CAPRISA, *n* = 3) were the most frequent institutions of these authors.

**Figure 4:**
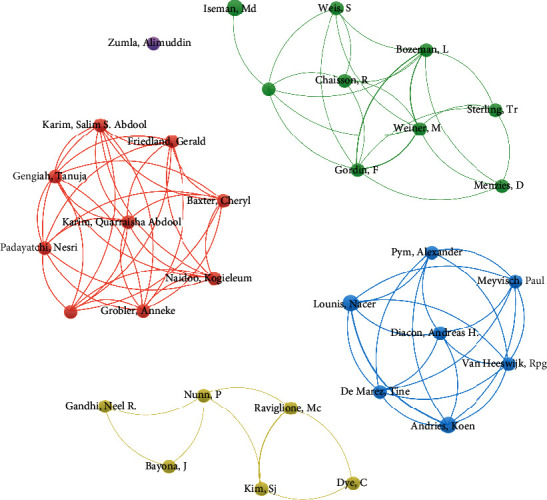
Collaborative network of authors (authors who have published more than 2 articles) of the top 50 cited literatures.

**Figure 5:**
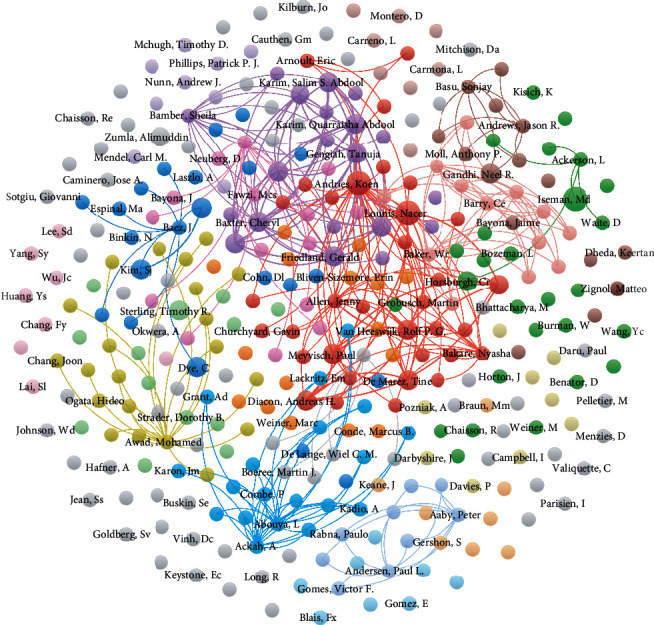
Collaborative network of all authors of the top 50 cited literatures.

Figure 4 shows the collaborative networks of the authors who have published more than 2 articles of the top 50 cited literatures.  Figure 5 shows the collaborative networks of all authors of the top 50 cited literatures. Each node represents an author; the links represent the association between authors; the color and distance between items represent the similarity between authors. The same color cluster had a strong cooperative relationship between these authors. It can be seen from the figure that the coauthors with more published articles are roughly divided into four relatively independent groups, while there is a complex correlation between other authors.

**Figure 6:**
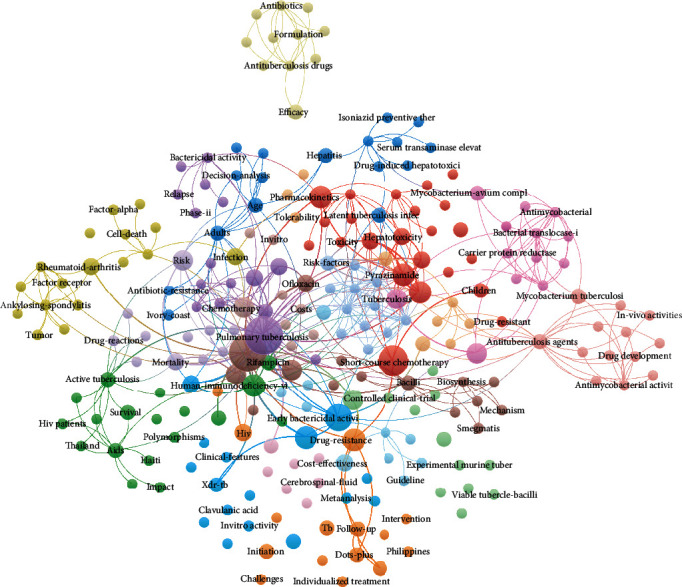
Cooccurrence network of keywords related to tuberculosis drug therapy.

**Figure 7:**
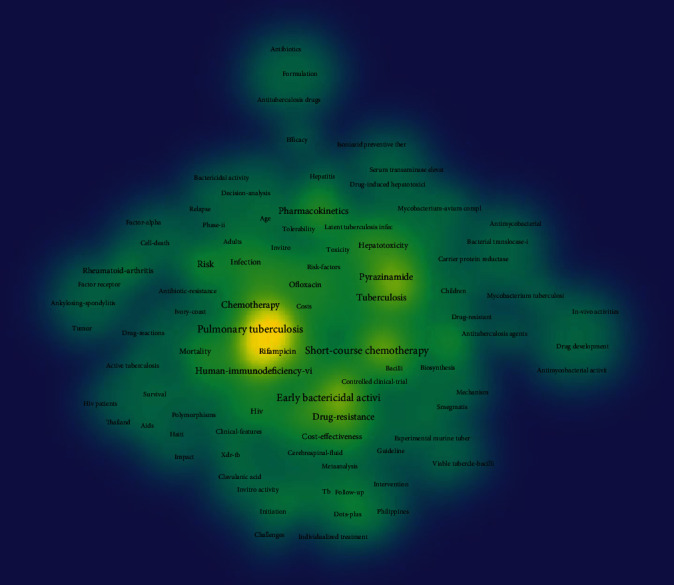
Cooccurrence density map of keywords related to tuberculosis drug therapy.

From the retrieved publications, keywords were extracted and cooccurrence frequencies were calculated. In total, 243 keywords were extracted and the network of keyword cooccurrence is shown in Figure 6. The size of the circles indicates the total frequency of occurrence for the keywords in the top 50 cited literatures. From the density map displayed in Figure 7, colors range from blue to green to yellow. The yellow area represents the research hotspots and directions in this field. The keywords mostly focused on pulmonary tuberculosis, etiology, drug resistance, tuberculosis complicated with HIV, treatment scheme selection, cost effectiveness and efficacy, epidemiology and transmission, and preventive therapy. Pulmonary tuberculosis, drug resistance, and treatment strategies turned out to be important topics.

## 4. Discussion

Among the diseases that are caused by a single pathogen, the disease burden of tuberculosis has been the highest for many years worldwide. Although the effectiveness of drug treatment for tuberculosis has been known for a long time, because of an increasing population flow, an increase in the human immunodeficiency virus (HIV) infection rate, irregular chemotherapy, treatment interruption, and other factors leading to the emergence of drug-resistant strains [75], tuberculosis is still prevalent throughout the world and cannot be eliminated in the short-term. Research has shown that among newly diagnosed patients, approximately 5% are drug-resistant tuberculosis patients [76]. The probability of coinfection with tuberculosis in patients with immune deficiency diseases within 10 years was 8%, and the mortality rate of these patients with a coinfection was as high as 30%, which was higher than the mortality rate of tuberculosis patients in the general population [77]. Therefore, the prevention and treatment of tuberculosis is still a great challenge. Drug treatment is still the main means to treat tuberculosis, and it would likely be the main treatment at present, even in the future for a long time [78]. Previously, bibliometric analysis literatures on tuberculosis [25, 26] did not focus on drug treatment, so we performed a study to evaluate research hotspots on tuberculosis drug therapy via top literatures.

The 50 top-cited literatures were published in 18 different journals between 1982 and 2014. The *New England Journal of Medicine* was the most frequent journal on our list, with 18 literatures (36%), followed by the *American Journal of Respiratory and Critical Care Medicine*, *Lancet*, *Lancet Infectious Diseases*, *Nature*, and *JAMA.*

This study found that the top-cited literatures were from many different countries, institutions, and authors. However, the USA showed a powerful influence, with approximately 60% of the literatures originating from institutions in the USA. Three of the five institutions that contributed more than two literatures were from the USA, among which the CDC and the American Psychological Society had a high influence in the field of tuberculosis treatment. The USA showed a strong connection with others and a great impact on other countries' research. In addition, the number of literatures from the WHO ranked highly, indicating that tuberculosis was a disease of global concern and that the WHO attached great importance to it. To understand the differences between the countries and institutions to which the corresponding authors and participating authors belong, we compared them. We found that there was no significant change in the results, but more participating authors came from different universities. The reason may be that most of the participating authors are school students.

The authors of the top-cited literatures on drug treatment of tuberculosis were located throughout the world. There were only two authors who contributed more than two literatures as first author. No corresponding author published more than one study. Possible reasons for this might include the following: first, tuberculosis had a wide range of influence, there were many institutions and researchers studying the disease, and these institutions and researchers were widely distributed; second, first-line oral antituberculosis drugs, including isoniazid, rifampicin, pyrazinamide, and ethambutol, had been commonly used internationally in the treatment of tuberculosis for many years [2], which lowered the threshold of research; and third, there were many kinds of drugs that could be used to treat tuberculosis, and they had different mechanisms, targets, and strategies [78], leading to more scattered cut-in points in research. Through the analysis of authors at different levels, it was found that the coauthors with more published articles are roughly divided into four relatively independent groups, while there is a complex correlation between other authors. In addition, South African authors have made greater contributions, which may be due to the high prevalence of tuberculosis in South Africa, which is an important research topic for their local health institutions.

Important achievements have been made in research on new antituberculotic drugs. In the last 50 years, the first new-mechanism antituberculotic drug, betaquiline, and the former nitroimidazole drug, delamanine, were approved for marketing. A variety of new mechanisms and new targets of antituberculosis candidates have entered the clinical stage [78]. For example, the nitroimidazole drug PA-824 [10] was in phase III clinical trials, and its mechanism of action involved inhibiting the synthesis of mycobacterial proteins and mycolic acid. The ethambutol drug SQ-109 was in phase II clinical trial, and its mechanism of action was inhibition of mycobacterium cell wall synthesis [79]. Combined with the visualization results, new drug treatments for drug-resistant tuberculosis and coinfection might be hot spots in future top-cited articles.

Our study also had some limitations. First, the citation analysis was based on the Web of Science Core Collection, which might miss some important literatures that were indexed by other databases, resulting in biased results. Second, searching using a topic search meant that a small number of manuscripts that involved drug treatment of tuberculosis might not have been identified. Third, this study excluded literatures that mentioned drug treatment of tuberculosis but that did not include tuberculosis as its main purpose of research, which were mainly reviews. Thus, it was possible that literatures with less content but a significant impact were missed. Fourth, since bibliometrics includes secondary research (such as review), the keywords and research focus of the secondary research may be different from the original research, which may lead to bias. Fifth, leading organizations that issue guidelines about TB treatment such as the CDC, Atlanta in the USA, and the WHO, Geneva in Switzerland, also add to the geographical bias. Sixth, VOS viewer software was used for visual analysis in this article. The definition of weight may be different from the actual frequency. At the same time, due to its limitations, it is unable to analyze more complex correlations.

In conclusion, our study identified the research hotspot of drug treatment of tuberculosis. Tuberculosis has a great burden of disease worldwide. Drug treatment of tuberculosis has been an important research field, and it will continue to be important now and in the near future. With the increasing incidence of drug-resistant tuberculosis, coinfection, and the emergence of new drugs, there will be increasing-impact drug treatment for tuberculosis-related research in the future.

## Figures and Tables

**Table 1:** The top 50 cited literatures on drug therapy for tuberculosis.

Title	Journal	Article type	Research contents/drugs	Total citation	Publication year	Page number	PMID
Tuberculosis associated with infliximab, a tumor necrosis factor (alpha)-neutralizing agent [27]	*New England Journal of Medicine*	Article	Infliximab	2689	2001	6	11596589
American Thoracic Society/Centers for Disease Control and Prevention/Infectious Diseases Society of America: treatment of tuberculosis [28]	*American Journal of Respiratory and Critical Care Medicine*	Review	Therapeutic monitoring	1380	2003	59	12588714
The emergence of drug-resistant tuberculosis in New York City [29]	*New England Journal of Medicine*	Article	Drug-resistant, rifapentine, isoniazid, HIV coinfection	786	1993	5	8381207
A small-molecule nitroimidazopyran drug candidate for the treatment of tuberculosis [30]	*Nature*	Article	Nitroimidazopyran	772	2000	4	10879539
The challenge of new drug discovery for tuberculosis [31]	*Nature*	Review	New drugs	757	2011	7	21270886
Multidrug-resistant and extensively drug-resistant tuberculosis: a threat to global control of tuberculosis [32]	*Lancet*	Article	Drug resistance	716	2010	13	20488523
Global surveillance for antituberculosis-drug resistance, 1994-1997 [33]	*New England Journal of Medicine*	Article	Drug resistance, isoniazid, rifampin, pyrazinamide, ethambutol	706	1998	8	9614254
An official ATS statement: hepatotoxicity of antituberculosis therapy [34]	*American Journal of Respiratory and Critical Care Medicine*	Review	Hepatotoxicity	675	2006	17	17021358
Treatment of 171 patients with pulmonary tuberculosis resistant to isoniazid and rifampin [35]	*New England Journal of Medicine*	Article	Drug resistance, isoniazid, rifampin	627	1993	5	8426619
The diarylquinoline TMC207 for multidrug-resistant tuberculosis [10]	*New England Journal of Medicine*	Article	TMC207, drug-resistant	590	2009	8	19494215
Anti-tumour necrosis factor agents and tuberculosis risk: mechanisms of action and clinical management [36]	*Lancet Infectious Diseases*	Review	Infliximab, etanercept	571	2003	7	12614731
Advances in the development of new tuberculosis drugs and treatment regimens [37]	*Nature Reviews Drug Discovery*	Review	New drug regimen	567	2013	16	23629506
Drug-therapy - treatment of multidrug-resistant tuberculosis [38]	*New England Journal of Medicine*	Review	Drug resistance	555	1993	7	8350889
Incidence of serious side effects from first-line antituberculosis drugs among patients treated for active tuberculosis [39]	*American Journal of Respiratory and Critical Care Medicine*	Article	Adverse reactions, isoniazid, rifampin, pyrazinamide, ethambutol	526	2003	5	12569078
Three months of rifapentine and isoniazid for latent tuberculosis infection [40]	*New England Journal of Medicine*	Article	Latent tuberculosis, rifapentine, isoniazid	514	2011	11	22150035
The effect of directly observed therapy on the rates of drug-resistance and relapse in tuberculosis [41]	*New England Journal of Medicine*	Article	Directly observed treatment, drug resistance	496	1994	5	8139628
Delamanid for multidrug-resistant pulmonary tuberculosis [11]	*New England Journal of Medicine*	Article	Delamanid (OPC-67683), drug-resistant	488	2012	9	22670901
Efficacy of various durations of isoniazid preventive therapy for tuberculosis - 5 years of follow-up in the IUAT trial [42]	*Bulletin of The World Health Organization*	Article	Isoniazid	484	1982	9	6754120
Effectiveness of recommendations to prevent reactivation of latent tuberculosis infection in patients treated with tumor necrosis factor antagonists [43]	*Arthritis and Rheumatism*	Article	Infliximab, latent tuberculosis	484	2005	6	15934089
Timing of initiation of antiretroviral drugs during tuberculosis therapy [44]	*New England Journal of Medicine*	Article, trail	Antiretroviral therapy, HIV coinfection	483	2010	9	20181971
Literatures on the treatment of tuberculosis undertaken by the British Medical Research Council Tuberculosis Units, 1946-1986, with relevant subsequent publications [45]	*International Journal of Tuberculosis and Lung Disease*	Review	Isoniazid, rifampicin, pyrazinamide, ethambutol, streptomycin	476	1999	48	10529902
Standard short-course chemotherapy for drug-resistant tuberculosis - treatment outcomes in 6 countries [46]	*JAMA-Journal of the American Medical Association*	Article	Drug-resistant, isoniazid, rifampicin, pyrazinamide, ethambutol, streptomycin	465	2000	8	10815117
Treatment of tuberculosis in patients with advanced human-immunodeficiency-virus infection [47]	*New England Journal of Medicine*	Article	HIV coinfection, isoniazid, rifampin, pyrazinamide, ethambutol	451	1991	5	1898769
Antituberculosis drugs: ten years of research [48]	*Bioorganic & Medicinal Chemistry*	Review	New drugs	439	2007	34	17291770
Short, highly effective, and inexpensive standardized treatment of multidrug-resistant tuberculosis [49]	*American Journal of Respiratory and Critical Care Medicine*	Article	Drug-resistant, gatifloxacin, clofazimine, ethambutol, pyrazinamide, prothionamide, kanamycin, isoniazid	436	2010	8	20442432
High-dose vitamin D-3 during intensive-phase antimicrobial treatment of pulmonary tuberculosis: a double-blind randomised controlled trial [50]	*Lancet*	Article	Vitamin D, supplementary	427	2011	8	21215445
Supplement – American Thoracic Society Centers for Disease Control and Prevention – targeted tuberculin testing and treatment of latent tuberculosis infection [51]	*American Journal of Respiratory and Critical Care Medicine*	Review	Latent tuberculosis infection, rifampin	415	2000	27	10764341
Antituberculosis drug-induced hepatotoxicity: concise up-to-date review [52]	*Journal of Gastroenterology and Hepatology*	Review	Hepatotoxicity, first-line drugs	410	2008	11	17995946
Chemotherapy and management of tuberculosis in the United Kingdom: recommendations 1998 [53]	*Thorax*	Review	Guidelines on chemotherapy	409	1998	12	9797751
Multidrug resistant pulmonary tuberculosis treatment regimens and patient outcomes: an individual patient data meta-analysis of 9,153 patients [54]	*Plos Medicine*	Review	Drug-resistant, fluoroquinolones, ethionamide, prothionamide	407	2012	0	22952439
Treatment outcomes among patients with multidrug-resistant tuberculosis: systematic review and meta-analysis [55]	*Lancet Infectious Diseases*	Review	Drug-resistant, treatment regimens	406	2009	8	19246019
WHO guidelines for the programmatic management of drug-resistant tuberculosis: 2011 update [56]	*European Respiratory Journal*	Article	Guidelines, drug-resistant	405	2011	12	21828024
Timing of antiretroviral therapy for HIV-1 infection and tuberculosis [57]	*New England Journal of Medicine*	Article	Antiretroviral therapy, HIV coinfection	403	2011	9	22010914
The potential advantages of nanoparticle drug delivery systems in chemotherapy of tuberculosis [58]	*American Journal of Respiratory and Critical Care Medicine*	Article	Nanoparticle-based drug delivery systems	403	2005	4	16151040
Effect of isoniazid prophylaxis on incidence of active tuberculosis and progression of HIV-infection [59]	*Lancet*	Article	Isoniazid, HIV coinfection	397	1993	4	8101302
Community-based therapy for multidrug-resistant tuberculosis in Lima, Peru [60]	*New England Journal of Medicine*	Article	Drug-resistant, pyrazinamide, ethambutol	392	2003	9	12519922
Multidrug-resistant tuberculosis and culture conversion with bedaquiline [61]	*New England Journal of Medicine*	Article	Bedaquiline (Sirturo, TMC207), drug-resistant	387	2014	10	25140958
Best drug treatment for multidrug-resistant and extensively drug-resistant tuberculosis [62]	*Lancet Infectious Diseases*	Article	Drug-resistant, multiple drugs	379	2010	9	20797644
Linezolid for treatment of chronic extensively drug-resistant tuberculosis [63]	*New England Journal of Medicine*	Article	Linezolid, drug-resistant	365	2012	11	23075177
Integration of antiretroviral therapy with tuberculosis treatment [64]	*New England Journal of Medicine*	Article	Integrating antiretroviral therapy, HIV coinfection	358	2011	10	22010915
Efficacy of trimethoprim-sulphamethoxazole prophylaxis to decrease morbidity and mortality in HIV-1-infected patients with tuberculosis in Abidjan, Cote d'Ivoire: a randomised controlled trial [65]	*Lancet*	Article	Trimethoprim-sulphamethoxazole, HIV coinfection	355	1999	7	10232312
Four-month moxifloxacin-based regimens for drug-sensitive tuberculosis [66]	*New England Journal of Medicine*	Article	Moxifloxacin, isoniazid, rifampin, pyrazinamide, ethambutol	355	2014	11	25196020
Toxic hepatitis with isoniazid and rifampin – a meta-analysis [67]	*Chest*	Review	Hepatotoxicity, isoniazid, rifampin	344	1991	7	1824929
Prospects for worldwide tuberculosis control under the WHO DOTS strategy [68]	*Lancet*	Article	Short-course drug regimen, HIV coinfection	342	1998	6	9863786
Polymorphism of the N-acetyltransferase 2 gene as a susceptibility risk factor for antituberculosis drug-induced hepatitis [69]	*Hepatology*	Article	Hepatotoxicity, isoniazid, genotyped NAT2	337	2002	7	11915035
A trial of three regimens to prevent tuberculosis in Ugandan adults infected with the human immunodeficiency virus [70]	*New England Journal of Medicine*	Article	First-line drugs, HIV coinfection	336	1997	8	9295239
Hepatotoxicity associated with isoniazid preventive therapy – a 7-year survey from a public health tuberculosis clinic [71]	*JAMA-Journal of the American Medical Association*	Article	Isoniazid, hepatotoxicity	336	1999	5	10086436
The magic bullets and tuberculosis drug targets [72]	*Annual Review of Pharmacology and Toxicology*	Review; book chapter	Chemotherapy review	322	2005	36	15822188
Rifapentine and isoniazid once a week versus rifampicin and isoniazid twice a week for treatment of drug-susceptible pulmonary tuberculosis in HIV-negative patients: a randomised clinical trial [73]	*Lancet*	Article	Rifapentine, isoniazid	318	2002	7	12241657
Vitamin D as supplementary treatment for tuberculosis a double-blind, randomized, placebo-controlled trial [74]	*American Journal of Respiratory and Critical Care Medicine*	Article	Vitamin D, supplementary	308	2009	8	19179490

**Table 2:** Journals that published the top 50 cited literatures.

Journal	Total citation	Number of study	Average citation	Impact factor (2019)
*New England Journal of Medicine*	10981	18	610	74.699
*American Journal of Respiratory and Critical Care Medicine*	4143	7	592	17.452
*Lancet*	2555	6	426	69.390
*Nature*	1529	2	765	42.779
*Lancet Infectious Diseases*	1356	3	452	24.446
*JAMA-Journal of the American Medical Association*	801	2	401	45.540
*Nature Reviews Drug Discovery*	567	1	567	64.797
*Bulletin of The World Health Organization*	484	1	484	6.960
*Arthritis and Rheumatism*	484	1	484	8.955
*International Journal of Tuberculosis and Lung Disease*	476	1	476	2.268
*Bioorganic & Medicinal Chemistry*	439	1	439	3.073
*Journal of Gastroenterology and Hepatology*	410	1	410	3.437
*Thorax*	409	1	409	10.844
*Plos Medicine*	407	1	407	10.500
*European Respiratory Journal*	405	1	405	12.339
*Chest*	344	1	344	8.308
*Hepatology*	337	1	337	14.679
*Annual Review of Pharmacology and Toxicology*	322	1	322	11.250

**Table 3:** Publishing years for the top 50 cited literatures.

Publication year	Number of study	Total citation	Average citation
1982	1	484	484
1991	2	795	398
1993	4	2365	591
1994	1	496	496
1997	1	336	336
1998	3	1457	486
1999	3	1167	389
2000	3	1652	551
2001	1	2689	2689
2002	2	655	328
2003	4	2869	717
2005	3	1209	403
2006	1	675	675
2007	1	439	439
2008	1	410	410
2009	3	1304	435
2010	4	2014	504
2011	6	2864	477
2012	3	1260	420
2013	1	567	567
2014	2	742	371

**Table 4:** Countries of the corresponding authors of the top 50 cited literatures.

Country	Number of literatures	Total citation	Average citation
USA	30	17515	584
Switzerland	4	1779	445
England	3	1312	437
Belgium	2	1193	597
Canada	2	1097	549
Spain	2	863	432
South Africa	2	841	421
France	1	439	439
Netherlands	1	410	410
Scotland	1	355	355
Cote d'Ivoire	1	355	355
China	1	337	337
Denmark	1	308	308

**Table 5:** Countries of all authors of the top 50 cited literatures.

Country	Weight of documents (≥3)
USA	31
England	10
South Africa	9
Switzerland	8
France	7
Peru	7
Canada	6
Netherlands	6
Russia	5
South Korea	5
Peoples R China	5
Belgium	4
Italy	4
Latvia	4
Spain	4
Philippines	3

**Table 6:** Institutions that published at least two of the corresponding author of 50 top-cited literatures.

Institution	Country	Number of study
Centers for Disease Control and Prevention	USA	4
WHO (World Health Organization)	Switzerland	3
ATS (American Thoracic Society)	USA	3
University of KwaZulu-Natal	South Africa	2
National Jewish Health Center	USA	2

**Table 7:** Institutions of all authors of the 50 top-cited literatures.

Institution	Country	Weight of documents (≥2)
Centers for Disease Control and Prevention	USA	7
WHO (World Health Organization)	Switzerland	5
McGill University	Canada	4
University College London	England	4
Columbia University	USA	3
International Union Against Tuberculosis and Lung Disease	—	3
Medical research council	England	3
University of California San Francisco	USA	3
University of Stellenbosch	South Africa	3
University of Washington	USA	3
Yale University	USA	3
Albert Einstein College of Medicine	USA	2
Boston University	USA	2
Harvard University	USA	2
Johns Hopkins University	USA	2
Korean Academy of Tuberculosis and Respiratory Diseases	Korean	2
Montefiore Medical Center	USA	2
National Masan TB Hospital	Korean	2
NIAID (National Institute of Allergy and Infectious Diseases)	USA	2
Tropical Disease Foundation	Philippine	2
University of KwaZulu-Natal	South Africa	2
Vanderbilt University	USA	2
Yonsei University	Korean	2

**Table 8:** Authors who published at least two literatures as first authors or corresponding authors.

Author	Name	Number of study	Institution	Country
First author	Diacon, Andreas H.	2	University of Stellenbosch	South Africa
	Karim, Salim S. Abdool	2	University of KwaZulu-Natal	South Africa

**Table 9:** Authors of the top 50 cited literatures.

Name	Weight of documents	Institution	Country
Andries, Koen	3	Janssen Research and Development	Belgium
Iseman, MD	3	National Jewish Center for Immunology and Respiratory Medicine	USA
Lounis, Nacer	3	Tibotec BVBA, Johnson & Johnson	Belgium
Baxter, Cheryl	2	The Centre for the AIDS Program of Research in South Africa (CAPRISA)	South Africa
De Marez, Tine	2	Janssen Research and Development	USA
Diacon, Andreas H.	2	University of Stellenbosch	South Africa
Dye, C	2	World Health Organization (WHO)	Switzerland
Friedland, Gerald	2	Yale University	USA
Gandhi, Neel R.	2	Albert Einstein College of Medicine	USA
Gengiah, Tanuja	2	The Centre for the AIDS Program of Research in South Africa (CAPRISA)	South Africa
Grobler, Anneke	2	University of Melbourne	Australia
Horsburgh, Cr	2	Boston University	USA
Karim, Quarraisha Abdool	2	University of KwaZulu-Natal	South Africa
Karim, Salim S. Abdool	2	University of KwaZulu-Natal	South Africa
Kim, Sj	2	Seoul Natl University	Korea
Meyvisch, Paul	2	Galapagos NV	Belgium
Naidoo, Kogieleum	2	University of KwaZulu-Natal	South Africa
Nair, Gonasagrie	2	The Centre for the AIDS Program of Research in South Africa (CAPRISA)	South Africa
Padayatchi, Nesri	2	University of KwaZulu-Natal	South Africa
Pym, Alexander	2	KwaZulu-Natal Research Institute for TB & HIV	South Africa
Raviglione, Mc	2	University of Milan	Italy
Sterling, Timothy R.	2	Vanderbilt University	USA
Van Heeswijk, Rolf P. G.	2	Janssen Infectious Diseases BVBA	Belgium
Zumla, Alimuddin	2	University College London	England

## Data Availability

The data used to support the findings of this study are included in the article. The original data can be retrieved on the Web of Science.

## References

[1] WHO Global tuberculosis report 2019. https://www.who.int/tb/publications/global_report/en/.

[2] Vos T., Flaxman A. D., Naghavi M. (2012). Years lived with disability (YLDs) for 1160 sequelae of 289 diseases and injuries 1990-2010: a systematic analysis for the Global Burden of Disease Study 2010. *The Lancet*.

[3] Naghavi M., Abajobir A. A., Abbafati C. (2017). Global, regional, and national age-sex specific mortality for 264 causes of death, 1980-2016: a systematic analysis for the Global Burden of Disease Study 2016. *The Lancet*.

[4] Bloom B. R., Murray C. J. (1992). Tuberculosis: commentary on a reemergent killer. *Science*.

[5] Frieden T. R., Sterling T. R., Munsiff S. S., Watt C. J., Dye C. (2003). Tuberculosis. *The Lancet*.

[6] Shabana S. M. A., Omar M. M., al mehy G. F., Mohammad O. E., Eldesouky R. S. (2015). Tuberculosis situation in Port Said governorate (1995-2011) before and after direct observed therapy short course strategy (DOTS). *Egyptian Journal of Chest Diseases and Tuberculosis*.

[7] Zhang Y., Heym B., Allen B., Young D., Cole S. (1992). The catalase--peroxidase gene and isoniazid resistance of *Mycobacterium tuberculosis*. *Nature*.

[8] Matteelli A., Carvalho A. C., Dooley K. E., Kritski A. (2010). TMC207: the first compound of a new class of potent anti-tuberculosis drugs. *Future Microbiol*.

[9] O'Brien R. J., Spigelman M. (2005). New drugs for tuberculosis: current status and future prospects. *Clinics in Chest Medicine*.

[10] Diacon A. H., Pym A., Grobusch M. (2009). The diarylquinoline TMC207 for multidrug-resistant tuberculosis. *The New England Journal of Medicine*.

[11] Gler M. T., Skripconoka V., Sanchez-Garavito E. (2012). Delamanid for multidrug-resistant pulmonary tuberculosis. *The New England Journal of Medicine*.

[12] Blair H. A., Scott L. J. (2015). Delamanid: a review of its use in patients with multidrug-resistant tuberculosis. *Drugs*.

[13] Blakeman K. (2018). Bibliometrics in a digital age: help or hindrance. *Science Progress*.

[14] Yeung A. W. K., Heinrich M., Atanasov A. G. (2018). Ethnopharmacology-a bibliometric analysis of a field of research meandering between medicine and food science?. *Frontiers in Pharmacology*.

[15] Tang H., Huang W., Ma J., Liu L. (2018). SWOT analysis and revelation in traditional Chinese medicine internationalization. *Chinese Medicine*.

[16] Feijoo J. F., Limeres J., Fernandez-Varela M., Ramos I., Diz P. (2014). The 100 most cited articles in dentistry. *Clinical Oral Investigations*.

[17] Yin X., Cheng F., Wang X. (2019). Top 100 cited articles on rheumatoid arthritis. *Medicine*.

[18] Yoon S. J., Yoon D. Y., Ja Lim K. (2019). The 100 top-cited articles focused on magnetic resonance: a bibliometric analysis. *Acta Radiol*.

[19] Kim H. J., Yoon D. Y., Kim E. S., Lee K., Bae J. S., Lee J. H. (2016). The 100 most-cited articles in neuroimaging: a bibliometric analysis. *Neuroimage*.

[20] Zhao X., Guo L., Lin Y. (2016). The top 100 most cited scientific reports focused on diabetes research. *Acta Diabetologica*.

[21] Nayar S. K., Dein E. J., Bernard J. A., Zikria B. A., Spiker A. M. (2018). Basic science research trends in orthopedic surgery: an analysis of the top 100 cited articles. *HSS Journal*.

[22] Zhang Z., Van Poucke S., Goyal H., Rowley D. D., Zhong M., Liu N. (2018). The top 2, 000 cited articles in critical care medicine: a bibliometric analysis. *Journal of Thoracic Disease*.

[23] Kreutzer J. S., Agyemang A. A., Weedon D. (2017). The top 100 cited neurorehabilitation papers. *NeuroRehabilitation*.

[24] Zhang Y., Quan L., Xiao B., Du L. (2019). The 100 top-cited studies on vaccine: a bibliometric analysis. *Human Vaccines & Immunotherapeutics*.

[25] Chen L. M., Liu Y. Q., Shen J. N. (2015). The 100 top-cited tuberculosis research studies. *The International Journal of Tuberculosis and Lung Disease*.

[26] Zhang Y., Huang J., Du L. (2017). The top-cited systematic reviews/meta-analyses in tuberculosis research. *Medicine*.

[27] Keane J., Gershon S., Wise R. P. (2001). Tuberculosis associated with infliximab, a tumor necrosis factor alpha-neutralizing agent. *The New England Journal of Medicine*.

[28] Blumberg H. M., Burman W. J., Chaisson R. E. (2003). American Thoracic Society/Centers for Disease Control and Prevention/Infectious Diseases Society of America: treatment of tuberculosis. *American Journal of Respiratory and Critical Care Medicine*.

[29] Frieden T. R., Sterling T., Pablos-Mendez A., Kilburn J. O., Cauthen G. M., Dooley S. W. (1993). The emergence of drug-resistant tuberculosis in New York City. *The New England Journal of Medicine*.

[30] Stover C. K., Warrener P., VanDevanter D. R. (2000). A small-molecule nitroimidazopyran drug candidate for the treatment of tuberculosis. *Nature*.

[31] Koul A., Arnoult E., Lounis N., Guillemont J., Andries K. (2011). The challenge of new drug discovery for tuberculosis. *Nature*.

[32] Gandhi N. R., Nunn P., Dheda K. (2010). Multidrug-resistant and extensively drug-resistant tuberculosis: a threat to global control of tuberculosis. *The Lancet*.

[33] Pablos-Méndez A., Raviglione M. C., Laszlo A. (1998). Global surveillance for antituberculosis-drug resistance, 1994-1997. World Health Organization-International Union against Tuberculosis and Lung Disease Working Group on Anti-Tuberculosis Drug Resistance Surveillance. *New England Journal of Medicine*.

[34] Saukkonen J. J., Cohn D. L., Jasmer R. M. (2006). An official ATS statement: hepatotoxicity of antituberculosis therapy. *American Journal of Respiratory and Critical Care Medicine*.

[35] Goble M., Iseman M. D., Madsen L. A., Waite D., Ackerson L., Horsburgh C. R. (1993). Treatment of 171 patients with pulmonary tuberculosis resistant to isoniazid and rifampin. *New England Journal of Medicine*.

[36] Gardam M. A., Keystone E. C., Menzies R. (2003). Anti-tumour necrosis factor agents and tuberculosis risk: mechanisms of action and clinical management. *The Lancet Infectious Diseases*.

[37] Zumla A., Nahid P., Cole S. T. (2013). Advances in the development of new tuberculosis drugs and treatment regimens. *Nature Reviews Drug Discovery*.

[38] Iseman M. D. (1993). Treatment of multidrug-resistant tuberculosis. *The New England Journal of Medicine*.

[39] Yee D., Valiquette C., Pelletier M., Parisien I., Rocher I., Menzies D. (2003). Incidence of serious side effects from first-line antituberculosis drugs among patients treated for active tuberculosis. *American Journal of Respiratory and Critical Care Medicine*.

[40] Sterling T. R., Villarino M. E., Borisov A. S. (2011). Three months of rifapentine and isoniazid for latent tuberculosis infection. *New England Journal of Medicine*.

[41] Weis S. E., Slocum P. C., Blais F. X. (1994). The effect of directly observed therapy on the rates of drug resistance and relapse in tuberculosis. *New England Journal of Medicine*.

[42] International Union Against Tuberculosis Committee on Prophylaxis (1982). Efficacy of various durations of isoniazid preventive therapy for tuberculosis: five years of follow-up in the IUAT trial. *Bulletin of the World Health Organization*.

[43] Carmona L., Gómez-Reino J. J., Rodríguez-Valverde V. (2005). Effectiveness of recommendations to prevent reactivation of latent tuberculosis infection in patients treated with tumor necrosis factor antagonists. *Arthritis & Rheumatism*.

[44] Abdool Karim S. S., Naidoo K., Grobler A. (2010). Timing of initiation of antiretroviral drugs during tuberculosis therapy. *New England Journal of Medicine*.

[45] Fox W., Ellard G. A., Mitchison D. A. (1999). Studies on the treatment of tuberculosis undertaken by the British Medical Research Council tuberculosis units, 1946-1986, with relevant subsequent publications. *The International Journal of Tuberculosis and Lung Disease*.

[46] Espinal M. A., Kim S. J., Suarez P. G. (2000). Standard short-course chemotherapy for drug-resistant Tuberculosis. *JAMA*.

[47] Small P. M., Schecter G. F., Goodman P. C., Sande M. A., Chaisson R. E., Hopewell P. C. (1991). Treatment of tuberculosis in patients with advanced human immunodeficiency virus infection. *The New England Journal of Medicine*.

[48] Janin Y. L. (2007). Antituberculosis drugs: ten years of research. *Bioorganic & Medicinal Chemistry*.

[49] van Deun A., Maug A. K., Salim M. A. (2010). Short, highly effective, and inexpensive standardized treatment of multidrug-resistant tuberculosis. *American Journal of Respiratory and Critical Care Medicine*.

[50] Martineau A. R., Timms P. M., Bothamley G. H. (2011). High-dose vitamin D_3_ during intensive-phase antimicrobial treatment of pulmonary tuberculosis: a double-blind randomised controlled trial. *The Lancet*.

[51] Cohn D., O'Brien R. J., Geiter L., Gordin F. M., Hershfield E., Horsburgh R. (2000). Targeted tuberculin testing and treatment of latent tuberculosis infection. *American Journal of Respiratory and Critical Care Medicine.*.

[52] Tostmann A., Boeree M. J., Aarnoutse R. E., de Lange W. C., van der Ven A. J., Dekhuijzen R. (2008). Antituberculosis drug-induced hepatotoxicity: concise up-to-date review. *Journal of Gastroenterology and Hepatology*.

[53] Joint Tuberculosis Committee of the British Thoracic Society (1998). Chemotherapy and management of tuberculosis in the United Kingdom: recommendations 1998. *Thorax*.

[54] Ahuja S. D., Ashkin D., Avendano M., Banerjee R., Bauer M., Bayona J. N. (2012). Multidrug resistant pulmonary tuberculosis treatment regimens and patient outcomes: an individual patient data meta-analysis of 9, 153 patients. *PLoS Medicine*.

[55] Orenstein E. W., Basu S., Shah N. S. (2009). Treatment outcomes among patients with multidrug-resistant tuberculosis: systematic review and meta-analysis. *The Lancet Infectious Diseases*.

[56] Falzon D., Jaramillo E., Schunemann H. J. (2011). WHO guidelines for the programmatic management of drug-resistant tuberculosis: 2011 update. *European Respiratory Journal*.

[57] Havlir D. V., Kendall M. A., Ive P. (2011). Timing of antiretroviral therapy for HIV-1 infection and tuberculosis. *The New England Journal of Medicine*.

[58] Gelperina S., Kisich K., Iseman M. D., Heifets L. (2005). The potential advantages of nanoparticle drug delivery systems in chemotherapy of tuberculosis. *American Journal of Respiratory and Critical Care Medicine*.

[59] Pape J. W., Jean S. S., Ho J. L., Hafner A., Johnson W. D. (1993). Effect of isoniazid prophylaxis on incidence of active tuberculosis and progression of HIV infection. *The Lancet*.

[60] Mitnick C., Bayona J., Palacios E. (2003). Community-based therapy for multidrug-resistant tuberculosis in Lima, Peru. *The New England Journal of Medicine*.

[61] Diacon A. H., Pym A., Grobusch M. P. (2014). Multidrug-resistant tuberculosis and culture conversion with bedaquiline. *The New England Journal of Medicine*.

[62] Caminero J. A., Sotgiu G., Zumla A., Migliori G. B. (2010). Best drug treatment for multidrug-resistant and extensively drug-resistant tuberculosis. *The Lancet Infectious Diseases*.

[63] Lee M., Lee J., Carroll M. W. (2012). Linezolid for treatment of chronic extensively drug-resistant tuberculosis. *The New England Journal of Medicine*.

[64] Abdool Karim S. S., Naidoo K., Grobler A. (2011). Integration of antiretroviral therapy with tuberculosis treatment. *The New England Journal of Medicine*.

[65] Wiktor S. Z., Sassan-Morokro M., Grant A. D. (1999). Efficacy of trimethoprim-sulphamethoxazole prophylaxis to decrease morbidity and mortality in HIV-1-infected patients with tuberculosis in Abidjan, Cote d'Ivoire: a randomised controlled trial. *The Lancet*.

[66] Gillespie S. H., Crook A. M., McHugh T. D. (2014). Four-month moxifloxacin-based regimens for drug-sensitive tuberculosis. *The New England Journal of Medicine*.

[67] Steele M. A., Burk R. F., Des Prez R. M. (1991). Toxic hepatitis with isoniazid and rifampin. A meta-analysis. *Chest*.

[68] Dye C., Garnett G. P., Sleeman K., Williams B. G. (1998). Prospects for worldwide tuberculosis control under the WHO DOTS strategy. *The Lancet*.

[69] Huang Y. S., Chern H. D., Su W. J. (2002). Polymorphism of theN-acetyltransferase 2 gene as a susceptibility risk factor for antituberculosis drug-induced hepatitis. *Hepatology*.

[70] Whalen C. C., Johnson J. L., Okwera A. (1997). A trial of three regimens to prevent tuberculosis in Ugandan adults infected with the human immunodeficiency virus. Uganda-Case Western Reserve University Research Collaboration. *The New England Journal of Medicine*.

[71] Nolan C. M., Goldberg S. V., Buskin S. E. (1999). Hepatotoxicity associated with isoniazid preventive Therapy. *JAMA*.

[72] Zhang Y. (2005). The magic bullets and tuberculosis drug targets. *Annual Review of Pharmacology and Toxicology*.

[73] Benator D., Bhattacharya M., Bozeman L. (2002). Rifapentine and isoniazid once a week versus rifampicin and isoniazid twice a week for treatment of drug-susceptible pulmonary tuberculosis in HIV-negative patients: a randomised clinical trial. *The Lancet*.

[74] Wejse C., Gomes V. F., Rabna P. (2009). Vitamin D as supplementary treatment for tuberculosis: a double-blind, randomized, placebo-controlled trial. *American Journal of Respiratory and Critical Care Medicine*.

[75] Wan Z. M., Xiang Y. G., Xiao-Hua M. A., Shi G. M., Fan R. H., Peng X. F. (2016). Advances in the mechanism of drug resistance to common first-line and second-line drugs in Mycobacterium tuberculosis. *Practical Preventive Medicine*.

[76] Gillette B. M., Jensen J. A., Tang B. (2008). *In situ* collagen assembly for integrating microfabricated three- dimensional cell-seeded matrices. *Nature Materials*.

[77] Healan A. M., Griffiss J. M., Proskin H. M. (2018). Impact of rifabutin or rifampin on bedaquiline safety, tolerability, and pharmacokinetics assessed in a randomized clinical trial with healthy adult volunteers. *Antimicrobial Agents and Chemotherapy*.

[78] Wang Y. M., Luan S. R., Zhou X. B. (2018). Research progress on anti-tuberculosis drugs. *Clinical Medication Journal*.

[79] Tahlan K., Wilson R., Kastrinsky D. B. (2012). SQ109 targets MmpL3, a membrane transporter of trehalose monomycolate involved in mycolic acid donation to the cell wall core of Mycobacterium tuberculosis. *Antimicrobial Agents and Chemotherapy*.

